# Detection of Epstein-Barr virus in recurrent tonsillitis

**DOI:** 10.1016/S1808-8694(15)30828-4

**Published:** 2015-10-18

**Authors:** Eliane Pedra Dias, Monica Lage da Rocha, Maria Odete de Oliveira Carvalho, Lidia Maria da Fonte de Amorim

**Affiliations:** 1 Doctorate in pathology, full professor of the Pathology Department, Medical School. Pathologist of the Pathological Anatomy Unit, Hospital Universitario Antonio Pedro. Coordinator of the graduation program in pathology - Universidade Federal Fluminense.; 2 Master’s degree in pathology - UFF. Dental surgeon, doctoral student in the pathology graduation program - focus on buccodental pathology, Universidade Federal Fluminense.; 3 Master’s degree in pathology, biologist.; 4 Doctorate in biology. Adjunct professor of the Cell and Molecular Biology Department, Instituto de Biologia da Universidade Federal Fluminense. Gratuate course in pathology, Hospital Universitário Antonio Pedro Universidade Federal Fluminense.

**Keywords:** immunohistochemistry, epstein-barr virus infection, pcr, tonsillitis

## Abstract

Recurrent tonsillitis has been the subject of frequent investigation. Misuse of antibiotic therapy in acute tonsillitis, changes to the tonsillar microflora, structural changes to the tonsillar crypts, and viral infections have been listed as predisposing or causal factors for recurrent tonsillitis. Epstein-Barr virus (EBV) infection usually occurs in early childhood and may persist in tonsillar lymphocytes, thus leading to the onset of recurrent tonsillitis. Little is known about the persistence and reactivation of EBV strains in immunocompetent patients. Methods such as in situ hybridization, polymerase chain reaction (PCR), and immunochemistry have been used to study the pathogenesis of the EBV. **Aim:** this study aims to characterize the association between EBV and recurrent tonsillitis by investigating the presence of EBV through PCR and immunohistochemistry, using viral protein LMP-1 as a target. **Study design:** this is a cross-sectional study with analysis of sample prevalence. **Materials and method:** twenty-four paraffin-embedded tonsil specimens from the Pathology Service were selected. The specimens were removed from children aged between 2 and 12 years diagnosed with recurrent tonsillitis. **Results:** EBV genome was detected in 13 (54.1%) specimens, whereas viral protein LMP-1 was found in 9 (37.5%) specimens. **Conclusion:** children’s tonsils can be colonized by EBV and such colonies may be associated with the pathogenesis of recurrent tonsillitis.

## INTRODUCTION

Waldeyer’s lymphatic ring is part of the first line of defense against pathogens; it is located at the entrance point of the airways and digestive tract. It consists of lymphoid tissue, which includes the palatine and pharyngeal tonsils. Many microorganisms may infect these tissues and cause tonsillitis; the most frequent are bacteria.^1^ The mechanism by which some children develop recurring tonsillitis is still unclear, as is the cause of exaggerated tonsillary enlargement. Many hypotheses have been raised, such as possible resistance of microorganisms to antibiotic therapy, and Streptococcus α interfering as an oropharyngeal protector against Streptococcus β to prevent recurring tonsillitis. Some studies have suggested that virus infections may be involved in recurring infections, including the Epstein-Barr virus (EBV) and herpes simplex viruses.^2^, ^3^, ^4^

The EBV was discovered in 1964 in a culture of Burkitt’s lymphoma cells; in 1968 studies revealed that it caused infectious mononucleosis.^5^ It is a herpesviridae of the subfamily gamaherpesvirinae, which infects most individuals before adult life. During the first infection, viruses are transmitted by saliva and invade the oropharyngeal epithelial cells, which are destroyed; it then infects circulating B lymphocytes, within which the virus becomes latent.^4^ The EBV genome consists of a linear 172 kilobase DNA molecule coding about 100 viral proteins; however, only 10 genes are expressed in vitro in infected B lymphocytes (latency). These 10 genes consist of six nuclear proteins (EBNAs 1, 2, 3A, 3B, 3C and EBNA-LP), two membrane proteins (LMP-1 and LMP-2), and two small RNAs (EBER 1 and EBER 2).^6^ The nuclear antigen protein 1 (EBNA-1) binds to the viral DNA so that the viral genome remains in infected cells as a circular episome; LMP-1 expression in immunocompromised individuals may induce B lymphocyte transformation and the onset of lymphoproliferative conditions.^6^

In contrast with in vitro studies, the EBV replication site has not been established in vivo. Infectious mononucleosis and oral hairy leukoplakia have been used as models for studying the replication mechanism of the virus.^3^ The EBV replication mode in healthy subjects is still unknown. The tonsils appear to be candidate sites for EBV replication.^7^, ^8^ Babcock (1998) detected linear episomal forms of EBV DNA in tonsillary lymphocytes. These linear forms suggest that virus DNA is replicating in tonsillary lymphocytes in healthy subjects previously infected by the EBV. Until recently it was thought that the virus was capable of infecting only B lymphocytes and epithelial cells; there have, however, been reports of infected normal T lymphocytes.^3^

The EBV is associated not only with infectious mononucleosis but also with other benign diseases, such as oral hairy leukoplakia, and malignancies, such as Hodgkin’s lymphoma, non-Hodgkin’s B and T cell lymphomas, and nasopharyngeal, gastric and breast carcinomas. It is currently being associated with autoimmune diseases, such as lupus erythematosus and multiple sclerosis.^5^, ^9^

Many molecular techniques are being used to demonstrate the presence of the EBV, such as the polymerase chain reaction (PCR) and in situ hybridization (IHS). PCR makes it possible to detect minimal amount of viral DNA in tissues and smears. According to Peiper (1990), PCR amplification of specific EBV genome sequences is a rapid, sensitive and specific method for identifying viral DNA. Furthermore, this method makes it possible to study biopsies that were paraffined and kept in files, which permits retrospective studies.

The purpose of this study was to investigate the association between the EBV and recurring tonsillitis by identifying the viral genome using PCR and the LMP-1 proteins by using immunohistochemistry.

## MATERIAL AND METHOD

A cross-sectional study was undertaken for analyzing the prevalence of EBV infection in tonsillectomy specimens, in which the indication for surgery had been recurrence and significant volume increase. The Research Ethics Committee of the Faculdade de Medicina (Medical School) - CEP-CMM approved this study (number 077/06). The slides of tonsillectomies with a diagnosis of hypertrophic chronic tonsillitis were selected among the tonsillectomies studied at the Pathology Unit from 1999 to 2006. After excluding inadequate cases (insufficient material, technical artifacts, inadequate fixation) 24 paraffined blocks with tonsils fixated in 10% buffered formaldehyde remained for study.

The nested PCR reaction using DNA extracted from three 5µm thickness tissue slices was used for identifying viral DNA. After sectioning each sample, the microtome was cleaned and the histological scalpels were changed. For DNA extraction the samples were deparaffinated in three xylene baths at 65ºC and hydrated with successive baths of 100%, 95% and 70% alcohol. After centrifugation, tissues were resuspended in 220µL of autoclaved milli-Q water and digested at 56ºC in the presence of 10% SDS (30µl) and K proteinase at 25mg% (6µL). K proteinase was added each day until the samples were completely digested. The suspension was extracted with phenol/chloroform/isoamylic alcohol (25:24:1) to remove undigested proteins, and after DNA precipitation with sodium acetate (3M) (35µL) and absolute alcohol (1mL), the samples were centrifuged and resuspended with sterile milli-Q water (50µL).^11^ Extracted genomic DNA quality and estimated quantity was verified using 1.7% agarose gel electrophoresis containing ethidium bromide. The genomic DNA of all samples underwent β-globin gene PCR (constitutive cell gene) to check whether the samples contained amplifiable DNA. The nested PCR reaction to amplify the EBNA-1 gene was used for identifying the EBV DNA, generating a 279 bp fragment in the first reaction and a 209 bp fragment in the second reaction.^12^ The first reaction mix, which identifies the herpex virus, contained 2.5µl of DNA from each extracted sample, 0.5µl of primers at 50µM (5’AAG-GAG-GGT-GGT-TTG-GAA-AG 3’ and 5’AGA-CAA-TGG-ACT-CCC-TTA-GC 3’), 2.5µl of a buffer solution (10mM TrisHCL pH8.3, 50mM of KCL), 0.75µl of MgCl at 2mM, 2µl dNTP at 2mM, and 0.2µl of Taq polymerase Invitrogen (5U/µl), and enough milli-Q water to reach a 25 µl end volume. The DNA used as a positive control was extracted from the EBV virus infected B9507 cell line; the negative control was done with sterile milli-Q water. Amplification was done using the following program: 2 minutes at 94ºC, followed by 35 30-second cycles at 94ºC, 30 seconds at 55ºC, and 30 seconds at 72ºC, followed by a 10 minute final extension at 72ºC. For the second reaction, specific primers for the EBV (5’ ATC-GTG-GTC-AAG-GAG-GTT-CC 3’ and 5’ ACT-CAA-TGG-TGT-AAG-ACG-AC 3’) were used under the same conditions with the following program: 45 seconds at 94ºC, followed by 35 20-second cycles at 94ºC, 30 seconds at 55ºC, 30 seconds at 72ºC, followed by a 10 minute final extension at 72ºC. Identification of gene amplification was done using an ethidium bromide stained 1.7% agarose gel, in which 7µl of each nested PCR product was separated by electrophoresis; a 100 bp molecular weight was used as the parameter (Fermentas).

To investigate the presence of the LMP-1 latency protein, paraffined blocks were microtomized and 5µm thickness sections were placed on pretreated slides with an adhesive (Silano®4%), after which deparaffining and washing with Tris-Buffered Saline was carried out. The slides were treated for antigen recovery during 30 minutes in a double boiler at 96ºC with a citrate buffer. The primary antibody was the monoclonal anti-LMP-1 (DAKO®) diluted at 1:400 according to the manufacturer’s instructions, and the kit LSAB (DAKO®) containing the biotinilated second antibody and the streptavidine and peroxidase conjugate. Revelation was done using a chromogenous solution (Kit DAB-DAKO®), and counterstaining was done with Harris’ hematoxyllin. Sections of lymphoma and inflammatory fibrous hyperplasia were used as positive and negative controls.

**Figure 1:**
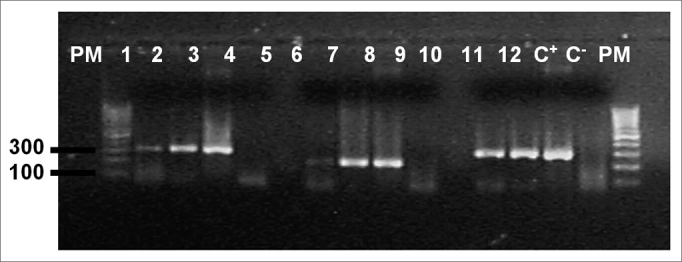
Agarose gel (1,7%) electrophoresis showing amplification of the β-globin gene, (268bp) PM - Molecular weight 100bp, Samples 1 - 12, C+ - DNA extracted from the line B9507, C- - Milli-Q water.

**Figure 2:**
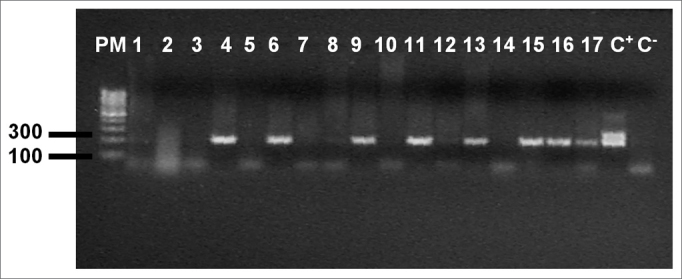
Agarose gel (1,7%) electrophoresis showing amplification of the EBV genome, (209bp) PM - Molecular weight 100bp, Samples 1 - 17, C+ - DNA extracted from the line B9507, C- - Milli-Q water.

**Figure 3:**
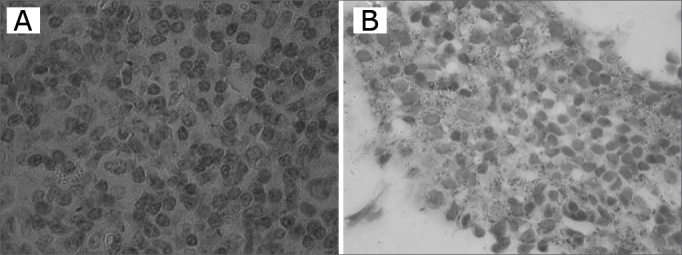
Immunohistochemistry showing positive results for the LMP-1 in cases A and B (1000X).

The genomic DNA visualized in agarose gel showed good quantity of DNA in extracted samples. Of 24 tonsils subjected to PCR, amplification of the β-globin gene was detected in 16 (66.7%) (Fig. 1). Of these, 10 (62.5%) amplified for the EBV genome (Table 1). EBV-DNA was detected in 13 (54.1%) of 24 tonsils (Fig. 2), of which 10 cases were from female patients (66.7% of 15 women) and 10 cases were from patients that comprised 58.8% of those aged from 6 to 12 years (Table 2). Investigation of the LMP-1 latency protein in 24 tonsils revealed immune reactivity in few lymphocytes of 9 tonsils (37.5%) (Fig. 3).

**Table 1:** Amplification of the β-globin gene and the EBV genome in tonsils.

	Tonsils	EBV +	EBV -
βglobin+	16	10(62,5%)	6(37,5%)
βglobin -	8	3(37,5%)	5(62,5%)

TOTAL	24	13(54%)	11(46%)

**Table 2:** Distribution of 24 tonsils in the sample according to the sex and age of patients, the indication for tonsillectomy, the histopathological diagnosis and positive results for the EBV-DNA and the LMP-1 protein.

Nº	Gender	Age	Tonsillectomy indication	Histopathology diagnosis	PCR -	LMP-1
1	M	10	PTAH	Hypertrophic chronic tonsillitis	-	-
2	F	6	PTAH	Hypertrophic chronic tonsillitis	-	-
3	F	9	PTAH & Repetition tonsillitis	Hypertrophic chronic tonsillitis	+	+
4	M	4	PTAH	Hypertrophic chronic tonsillitis	-	-
5	M	6	PTAH	Hypertrophic chronic tonsillitis	+	+
6	F	7	PTAH & Repetition tonsillitis	Hypertrophic chronic tonsillitis	+	+
7	M	6	Repetition tonsillitis	Hypertrophic chronic tonsillitis	-	-
8	M	10	PTAH	Hypertrophic chronic tonsillitis	+	+
9	F	9	PTAH	Hypertrophic chronic tonsillitis	+	-
10	F	12	PTAH	Hypertrophic chronic tonsillitis	+	-
11	M	6	PTAH & Repetition tonsillitis	Hypertrophic chronic tonsillitis	-	-
12	F	12	PTAH & Repetition tonsillitis	Hypertrophic chronic tonsillitis	-	-
13	M	5	PTAH	Hypertrophic chronic tonsillitis	-	-
14	M	5	Repetition tonsillitis	Hypertrophic chronic tonsillitis	+	-
15	F	12	PTAH	Hypertrophic chronic tonsillitis	-	-
16	F	8	PTAH	Hypertrophic chronic tonsillitis	-	-
17	F	6	PTAH	Hypertrophic chronic tonsillitis	+	+
18	M	3	PTAH	Hypertrophic chronic tonsillitis	-	-
19	F	4	PTAH & Repetition tonsillitis	Hypertrophic chronic tonsillitis	+	-
20	F	12	PTAH	Hypertrophic chronic tonsillitis	+	+
21	F	10	PTAH	Hypertrophic chronic tonsillitis	+	+
22	F	2	PTAH	Hypertrophic chronic tonsillitis	+	+
23	F	4	PTAH	Hypertrophic chronic tonsillitis	-	-
24	F	10	PTAH	Hypertrophic chronic tonsillitis	+	+

Nº = Case number PTAH=pharyngeal tonsil and adenoid hypertrophy + positive - negative

## RESULTS

The sample used in this study originated from pediatric patients aged from 2 to 12 years (mean 7.4 years, standard deviation 3.1 years), of which 15 (62.5%) were female and 9 (37.5%) were male. Tonsillectomy was indicated due to tonsillary hypertrophy and adenoid vegetations in 17 cases, due to tonsillary hypertrophy and adenoid vegetations and recurring tonsillitis in 5 cases, and due to recurring tonsillitis in 2 cases.

## DISCUSSION

The Epstein-Barr virus causes infectious mononucleosis and oral hairy leukoplakia; it has also been associated with numerous malignancies including Hodgkin’s disease, B and T lymphomas, nasopharyngeal carcinomas, and gastric carcinomas in immunosuppressed patients. Little is known, however, about the pathogeny of the virus in immunocompetent patients. Many researchers have suggested that the tonsils are a possible replication site for this virus.^2^, ^3^, ^8^, ^13^

Molecular techniques have been used often for diagnosing and monitoring patients with virus diseases. The EBV-DNA can be identified by in situ hybridization (ISH) and the polymerase chain reaction (PCR); some authors consider both equally sensitive for detecting the EBV.^14^ Additionally, viral proteins found in latent and replicative infection may be identified by immunohistochemical techniques.^2^, ^3^, ^4^, ^13^ The tonsils are considered the site of initial infection, and of viral persistence and replication. The EBV may infect the tonsils of children and become involved in recurring tonsillitis.^2^, ^15^, ^16^

In our study we selected the tonsils of children with a mean age of 7,4 years and a diagnosis of chronic and hypertrophic tonsillitis. We believe that the significant number of recurring tonsillary infections in childhood may be associated with the EBV; initial contact with this virus often occurs around age 7 years. Sixteen of 24 tonsils showed positive amplification for β-globin, thus demonstrating the presence of constitutional DNA, which was amplifiable, in the material that was extracted. In 10 of these 16, amplification of EBV was obtained, as in three tonsils that were negative for β-globin, suggesting that, although EBV-DNA was amplified in these three samples, we cannot state that the other five EBV-negative samples did not amplify by lack of the virus or low quantity or quality of DNA. It can be stated that the EBV infected 54.1% of the tonsils in our sample; but mapping this infection, which would make it possible to identify the target cell population, faces technical limits. Niedobitek et al.^17^ used the ISH to define the pattern and distribution of EBV-positive lymphoblasts found mostly in extrafollicular areas. The prevalence of EBV infection in tonsillitis varies according to the detection method. Studies using the ISH for detecting EBER found a 26%,19 a 29%,2 and a 65%4 association of the EBV with tonsillitis. Other studies using the PCR found a prevalence of up to 65% (11%,^20^ 58%,^15^ and 64%^17^). The PCR thus confirms its higher sensitivity for molecular detection, and suggests an association between the EBV and tonsillitis.

Ping-Ching Pai et al.’s^15^ results using nested PCR to investigate an association between the EBV and 57 tonsillitis cases (58%) and 31 tonsils with malignancies (51%) suggest that the association is not restricted only to recurring tonsillitis.

Ikeda et al.^3^ (2000) used the ISH, the RT-PCR, and immunohistochemistry in 15 fragments of tonsils from patients with chronic tonsillitis. In a detailed study, these authors showed that tonsillary lymphocytes are not only a reservoir but also a replication environment for the EBV. Our results similarly found the LMP-1 (the EBV latency protein) in 37.5% of tonsils in our sample, showing that immunohistochemical methods may be an initial investigation method for latent EBV infection. Many question remain to be studies in the pathogeny of EBV infection; its association with the tonsils is particularly challenging.

## CONCLUSION

Identification of a high (54.1%) prevalence of EBV-DNA and identification of the LMP-1 (37.5%) in recurring tonsillitis in children suggest that the tonsils may be reservoir for the EBV, and that this virus may be involved in recurring infection. Many aspects of latent and replicative EBV infection remain unclear, and are possible points for future research.
